# Association of Gene Expression Profiles in HPV-Positive Head and Neck Squamous Cell Carcinoma with Patient Outcome: In Search of Prognostic Biomarkers

**DOI:** 10.3390/ijms26125894

**Published:** 2025-06-19

**Authors:** J. Noé García-Chávez, Adriana Contreras-Paredes, Claudia González-Espinosa, Imelda Martínez-Ramírez, Elizabeth Langley, Marcela Lizano, J. Omar Muñoz-Bello

**Affiliations:** 1 Unidad de Investigación Biomédica en Cáncer, Instituto Nacional de Cancerología, Mexico City 14080, Mexico; jorge.garcia@cinvestav.mx (J.N.G.-C.); acontrerasp@incan.edu.mx (A.C.-P.); imartinezr@incan.edu.mx (I.M.-R.); langleyemx@gmail.com (E.L.); 2 Departamento de Farmacobiología y Centro de Investigación Sobre el Envejecimiento, Centro de Investigación y de Estudios Avanzados, Unidad Sede Sur, Mexico City 14330, Mexico; cgonzal@cinvestav.mx; 3 Departamento de Medicina Genómica y Toxicología Ambiental, Instituto de Investigaciones Biomédicas, Universidad Nacional Autónoma de Mexico, Ciudad Universitaria, Mexico City 04510, Mexico

**Keywords:** human papillomavirus, biomarkers, HNSCC, transcriptomic analysis

## Abstract

Head and Neck Squamous Cell Carcinoma (HNSCC) ranks sixth in incidence and seventh in cancer mortality worldwide. Approximately 30% of HNSCC cases are related to human papillomavirus (HPV) infection, the oropharynx being the anatomical subsite most associated with HPV infection. Traditionally, HPV-positive HNSCC has been considered to have better treatment response and clinical outcome. However, HPV-positive HNSCC is a heterogeneous group since 30% of the cases present early relapse, which implies that there are differences in molecular profiles within HPV-positive patients. In this study, we used bioinformatic data analysis from open-access repositories to compare molecular profiles differentially expressed between HPV-positive and -negative HNSCC patients. Using the TCGA HNSCC transcriptomic data, we identified a group of genes, whose expression is related to clinical outcome in patients. Our findings were validated in an independent cohort confirming that the expression levels of *FABP4*, *HMGA2*, *S100A10*, *GDNF*, *SLC7A,2* and *GPR18* genes were associated with overall survival (OS) exclusively in HPV-positive HNSCC patients, while *ST6GALNAC1* expression was associated with OS in HPV-negative HNSCC. The expression of OS-related genes was independent of tumor stage and history of alcoholism. Our findings suggest that transcriptional profiles in HPV-positive HNSCC are an excellent source of information for the search for potential prognostic biomarkers.

## 1. Introduction

Head and Neck Squamous Cell Carcinoma (HNSCC) arises in the squamous cells covering the tissues of the head and neck region and comprises different anatomical subsites, including lips and oral cavity salivary glands, nasopharynx, oropharynx, hypopharynx, and larynx [1]. The sum of all the cases of the anatomical subsites that are grouped in HNSCC ranks it seventh in incidence and sixth in cancer mortality worldwide, with an estimated total of 947,211 and 482,428 new cases in incidence and mortality, respectively, in 2022 [2]. Different risk factors have been associated with the development of HNSCC, including tobacco [3] and alcohol consumption [4], betel nut chewing [5], and persistent human papillomavirus (HPV) infection [6].

Close to 30% of all HNSCC cases are HPV-positive [7,8], and according to the anatomical subsite, most HPV-positive HNSCCs correspond the oropharynx (45.8%), while 22.1% and 24.2% develop in the larynx (including hypopharynx) and oral cavity, respectively [8]. In HNSCC, the most prevalent viral type is HPV-16 in 86% of the cases, followed by HPV-18 in 27%, while the co-infection of both viral types has been reported in 12% [9]. Between 1988 and 2004, there was an alarming 225% increase in the number of HPV-associated Oropharyngeal Squamous Cell Carcinoma (OPSCC) cases, while the number of HPV-negative patients decreased by 50% [10].

The transforming capacity of HPV is attributed to the continuous expression of viral oncoproteins E6 and E7, which prevent apoptosis and induce cell cycle continuity. Most HPV infections are transient and are eliminated by the immune system. However, if these infections persist, the HPV episome breaks down over time and integrates randomly into the host genome. When the viral genome breaks within the E2 gene, this promotes the over-expression of E6 and E7 oncoproteins, leading to the development of cancer [11].

HPV-positive HNSCC represents a distinct tumor entity that differs in cell biology and clinical course from HPV-negative tumors [12]. Moreover, the presence of HPV is considered a determining factor in the clinical outcome of patients [13]. For instance, patients with HPV-positive OPSCC exhibit a 5-year overall survival (OS) rate of 81.1%, compared with 39.7% for HPV-negative tumors [14]. Even though HPV-positive HNSCC is associated with better OS, currently there are no differences in treatments for patients with HPV-positive and -negative tumors, indicating a need to analyze the possibility of de-intensifying treatments to improve quality of life. While the standard 7-week chemotherapy and radiotherapy treatment for HPV-associated OPSCC is effective, it produces adverse effects in patients, presenting acute complications (such as mucositis, dermatitis, dysphagia, odynophagia, alopecia) or late complications (skin changes, xerostomia, trismus, dental caries, swallowing dysfunction, lymphedema). Eventually, these complications lead to treatment discontinuation, affecting patients’ quality of life [15,16].

Recently, our group conducted a comprehensive literature search for potential transcript-based prognostic biomarkers that predict clinical outcomes in patients with HPV-positive HNSCC [17], since prognostic biomarkers in cancer indicate the clinical evolution of patients to achieve individualized treatment or to stratify patients with a higher risk of death, recurrence, or progression [18,19]. Open-access database repositories such as The Cancer Genome Atlas Program (TCGA) and Gene Expression Omnibus (GEO) offer an invaluable opportunity to investigate transcriptional profiles in HPV-associated tumors, including HNSCC. Such databases contain clinical–pathological information on patients, allowing the analysis of gene expression changes and the association of molecular profiles with patient clinical outcomes. Massive RNA sequencing has become a fundamental tool for the identification of transcriptional profiles in cancer, which has allowed the discovery of potential biomarkers associated with the evolution of the disease, and the investigation of molecular aspects inherent to the tumor, such as heterogeneity, tumor microenvironment, and resistance to therapy, among others. A number of studies highlight the utility of transcripts as prognostic biomarkers in various tumor types, including HNSCC [17,20,21,22,23].

Due to the constant increase in new cases and deaths in HNSCC tumors, particularly in those positive for HPV, it is imperative to identify biomolecules that predict a higher risk of recurrence and low survival rate, allowing for closer clinical follow-up and providing a basis for informed choices for appropriate therapeutic strategies. In this work, we aimed to identify differentially expressed mRNAs in HPV-positive HNSCC compared to HPV-negative tumors, finding those transcripts that impact OS exclusively in patients with HPV-positive HNSCC, highlighting a set of genes associated with clinical outcome and related to a molecular phenotype unique to HPV-positive cases.

## 2. Results

### 2.1. Selection of TCGA HNSCC Samples According to HPV-Status for the Discovery Cohort

To provide greater accuracy in this study, we included samples that were annotated as HPV-positive (final HPV status) in the HNSCC cohort from the TCGA database, as well as those that were re-classified as HPV-positive in previous omics analyses [24,25,26]. Therefore, 61 samples were included that were considered to be truly HPV-positive, while 409 samples were considered truly HPV-negative. The clinicopathological features of our cohort were analyzed and are shown in Supplementary Table S1.

### 2.2. Differential Expression Profiles in HPV-Positive HNSCC and Their Potential Implication in Biological Processes, Molecular Functions, and Signaling Pathways

**Figure 1:**
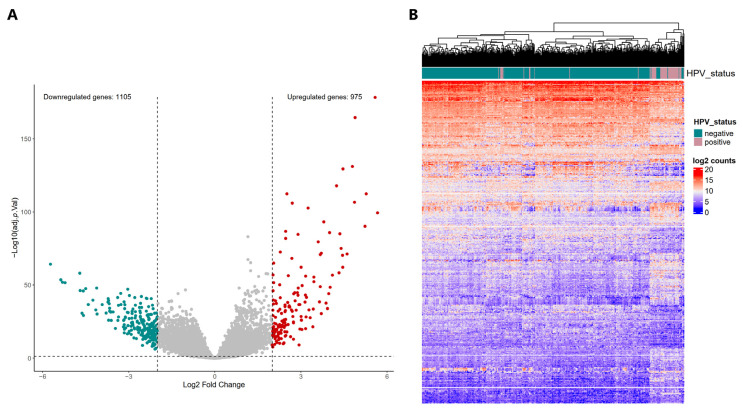
Gene expression profiles in HPV-positive HNSCC. (**A**) Volcano plot shows the log2 fold change in differentially expressed genes and adjusted *p* value (adj. *p*. Val) in −log10 scale. Up-regulated genes are shown in red, and down-regulated genes are shown in green. (**B**) Heatmap of the expression levels (log2 counts) of the differentially expressed genes in the TCGA HNSCC cohort. The dendrogram shows that HPV-positive samples (pink color) are mostly grouped on the right and HPV-negative samples on the left (green).

The differential expression analysis includes only genes with 10 or more counts in 90% of the samples in at least one of the groups (HPV-positive or HPV-negative). A total of 18,464 genes were included for differential expression analysis (DEA). We identified 2080 differentially expressed genes (DEGs) in HPV-positive HNSCC when compared to HPV-negative cases. Of these, 1105 genes were down-regulated and 975 were up-regulated (Figure 1) (Supplementary Table S2).

**Figure 2:**
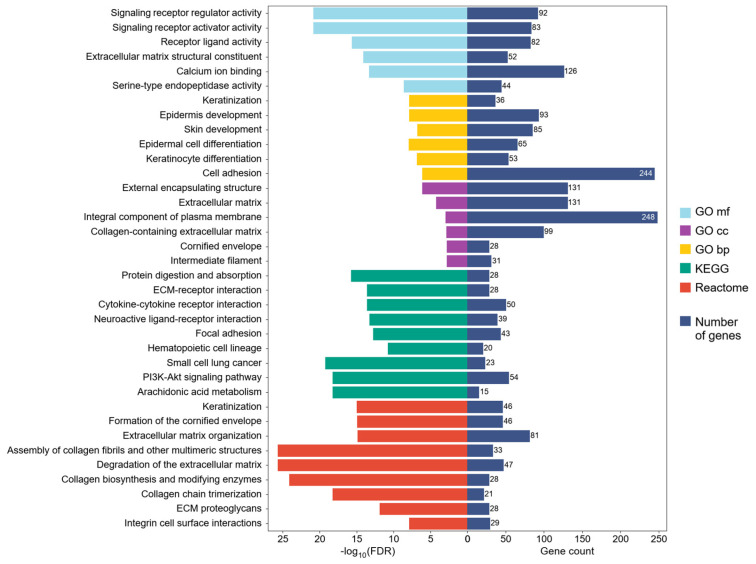
GO terms, Reactome, and KEGG pathway enrichment analysis. The top GO terms and pathways are shown in the figure according to their enrichment and negative base-10 logarithm of false discovery rate (FDR).

We found that over-represented pathways of DEGs were associated with protein digestion and absorption, arachidonic acid metabolism, PI3K-Akt signaling, extracellular matrix (ECM) organization, ECM proteoglycans, ECM–receptor interaction, cytokine–cytokine receptor interaction, integrin cell surface interactions, focal adhesion, keratinization, the formation of cornified envelope, the assembly of collagen fibrils and other multimeric structures, and collagen chain trimerization. Over-represented gene ontology (GO) terms include similarities with signaling pathways related to ECM, keratinization, and skin and epidermis development and differentiation. Over-represented molecular function GO terms include serine-type endopeptidase activity, calcium ion binding, and extracellular matrix structural constituent. GO terms related to biological processes including cell adhesion, keratinization, epidermis and skin development, and the differentiation of keratinocytes and epidermal cells were enriched in HPV-positive HNSCC. Over-represented cellular component GO terms were external encapsulating structure, ECM, components of the cellular membrane, collagen, cornified envelope, and intermediate filament (Figure 2). The complete list of over-represented GO terms is shown in Supplementary Table S3, where the Kyoto Encyclopedia of Genes and Genomes (KEGG) and Reactome pathways are also presented.

### 2.3. HPV Positivity of HNSCC Is Associated with Improved Overall Survival

**Figure 3:**
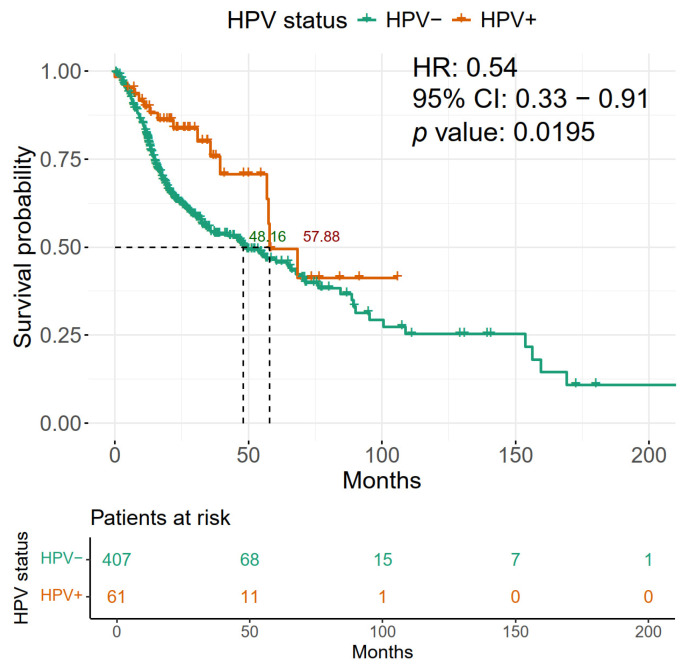
Patients with HPV-positive HNSCC exhibit a longer overall survival than HPV-negative patients. HPV-positive cases are shown in the red line, HPV-negative in the green line. The mean survival probability of each group is shown in dotted lines. The time scale is shown in months. The Cox proportional hazard model was performed with the survival package in R and survival data are represented in Kaplan–Meier plots. The dotted line represents the 50% probability of survival.

To evaluate the impact of HPV presence on OS in patients with HNSCC in our discovery cohort (TCGA), a survival curve analysis was performed comparing HPV-positive and -negative patients. We found that HPV-positive patients had an improved OS with 57.88 months vs. 48.16 months for HPV-negative patients (Figure 3). Furthermore, the presence of HPV in these tumors shows a hazard ratio (HR) of 0.54, which offers greater protection for the OS of patients.

### 2.4. Differentially Regulated Genes in HPV-Positive HNSCC Show an Association with Patient Overall Survival

To gain insight into the survival behavior of HPV-positive patients and identify a subgroup of patients with a poor prognosis, we evaluated whether the 18,464 genes that were included in the DEA only impact this behavior in the group of HPV-positive HNSCC patients, using the TCGA data as our discovery cohort. Each of these genes was dichotomized into high and low expression based on median, and survival curves were generated to assess their impact on clinical outcomes. We identified 1648 genes that were significantly associated with OS (univariate Cox regression and log rank *p* value < 0.05); of these, 819 had a high expression with a protective effect (95% confidence interval < 1) and 829 had a high expression that resulted in poor prognosis (95% confidence interval > 1) (Supplementary Table S4). Among these genes, 160 were differentially expressed in HPV-positive HNSCC patients compared with HPV-negative patients. Therefore, we considered these genes to be putative biomarkers for HPV-positive HNSCC. Then, to validate the findings of our discovery cohort, we evaluated the prognostic impact of the expression levels of the previously detected 2080 DEGs (Figure 1A) using the GSE65858 HNSCC dataset as a validation cohort [27]. Of these genes, 108 were related to OS (Supplementary Table S4). After analyzing the discovery and validation cohorts, we identified that eight genes (*S100A10*, *FABP4*, *HMGA2*, *SLC7A2*, *GPR18*, *PPP1R1C*, *GDNF*, and *MAP2*) were associated with OS in HPV-positive HNSCC patients in both cohorts (*p* value < 0.05) (Supplementary Table S5). Importantly, these eight genes found in both the discovery and validation cohorts were consistent in their HR, either as protective or as having a high risk of death. Furthermore, only genes associated with OS that were altered solely in HPV-positive tumors were retained.

We used a similar approach to evaluate the association of gene expression levels with OS in HNSCC HPV-negative patients. We identified 1538 genes whose higher expression levels are associated with OS (*p* value < 0.05, univariate Cox analysis) in the discovery cohort. Of these, 734 were associated with higher risk (95% confidence interval > 1), and 804 with a protective effect (95% confidence interval < 1) (Supplementary Table S4). Among these genes, 222 were differentially expressed in the discovery cohort when comparing HPV-positive to HPV-negative HNSCC. Therefore, these were considered as putative biomarkers in HPV-negative HNSCC patients. Additionally, when analyzing the validation GSE65858 dataset, we identified 192 genes whose expression levels were associated with OS (*p* value < 0.05) in HPV-negative patients (Supplementary Table S4). Of these genes, 13 (*ALOX12B*, *CRYM*, *CXCL17*, *DLX3*, *FAM25A*, *FUT6*, *IVL*, *KRT6C*, *SPRR2A*, *SPRR4*, *ST6GALNAC1*, *TJP3*, and *ZNF541*) were related to OS in both the discovery and the validation datasets (Supplementary Table S5). Notably, the 13 genes associated with OS in HPV-negative HNSCC showed concordance in HR, either as a protective factor or as a high-risk factor, in both the discovery and validation cohorts. Only genes that showed association with OS in HPV-negative tumors were retained.

In total, we identified 21 differentially expressed genes between HPV-positive and HPV-negative HNSCC, which were associated with OS, 13 associated with HPV-negative, and 8 related to HPV-positive HNSCC patients (Supplementary Table S5).

### 2.5. Confounding Factors Analysis Using Multivariate Cox Models Reveals Independent Biomarkers for HPV-Positive and HPV-Negative HNSCC Patients

We then examined whether disease stage and alcohol history were confounders of clinical outcome. We used multivariate Cox proportional hazard models, including the gene expression levels, the stage of the disease, and alcohol consumption in the analysis (see Section 4). The discovery and validation cohorts were evaluated independently. Herein, we only show those genes that were significantly associated with OS (*p* value < 0.05, Cox proportional hazard model and log rank test) and not significantly associated with any of the confounders (*p* value > 0.05, Cox proportional hazard model). Thus, after the independent analysis of the discovery and validation cohorts, we confirmed that 7 genes were not significantly associated with disease stage or alcohol consumption history, but their expression levels showed a significant relationship with the prognosis of patients with HNSCC (Table 1). We considered these genes to be independent prognostic biomarkers for patients with HNSCC.

### 2.6. The Expression of Candidate Biomarker Genes Is Associated with the Clinical Outcome of Patients with HNSCC in the Discovery and Validation Cohorts

**Figure 4:**
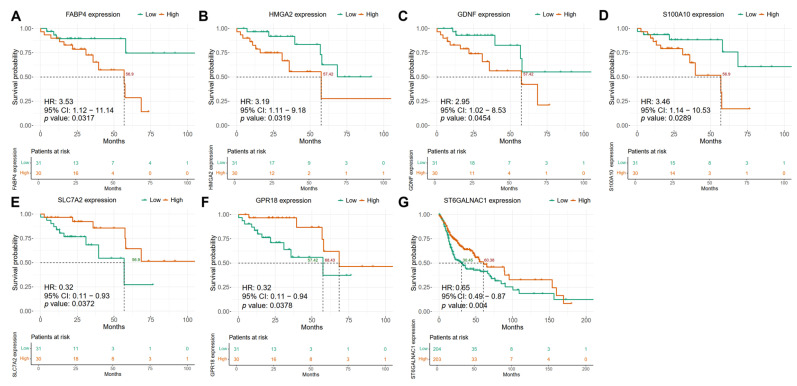
Genes associated with overall survival in HPV-positive HNSCC patients. Expression levels of (**A**) *FABP4*, (**B**) *HMGA2*, (**C**) *GDNF*, (**D**) *S100A10*, (**E**) *SLC7A2*, (**F**) *GPR18*, and (**G**) *ST6GALNAC1* genes were dichotomized into high (red line) and low (green line) in HNSCC patients and Kaplan–Meier analyses were performed to determine OS. The median survival probability is shown in dotted lines. The time scale is represented in months. Univariate Cox proportional hazard model was performed with a survival package in R and survival data were represented in the Kaplan–Meier plots. The dotted line represents the 50% probability of survival.

In the HPV-positive HNSCC group of the discovery cohort, Kaplan–Meier survival analysis revealed that high expression levels of *FABP4*, *HMGA2*, *GDNF*, and *S100A10* genes were associated with significantly worse overall survival (OS) (Cox regression and log rank *p* value < 0.05, HR > 2.9, 95% lower confidence interval > 1) (Supplementary Table S5) (Figure 4A–D). For example, the high expression of *FABP4* was associated with a reduced mean OS of 56.9 months, whereas the survival curve for the low-expression group did not fall below the 0.5 survival probability threshold (Figure 4A). Similar trends were observed for *HMGA2*, *GDNF*, and *S100A10*, with mean OS values for the high-expression groups ranging from 56.9 to 57.42 months, whereas the corresponding low-expression groups failed to reach the median OS determined in the study (Figure 4B–D). Conversely, high expression of gene *SLC7A2* was associated with a protective effect, with a mean OS of 56.9 months (Cox regression and log rank *p* value 0.0372, HR 0.32, 95% confidence interval higher range < 1), while the low-expression group did not reach the 0.5 survival probability threshold (Supplementary Table S5) (Figure 4E). Similarly, the high expression of *GPR18* was linked to improved survival compared to the low-expression group (68.43 vs. 57.42 months) (Cox regression and log rank *p* value 0.0378, HR 0.32, 95% confidence interval higher range < 1) (Figure 4F) (Supplementary Table S5). In HPV-negative HNSCC patients, we observed that the high expression of *ST6GALNAC1* is associated with better prognosis, as the mean OS probability is greater than in the low-expression subgroup (Cox regression and log rank *p* value 0.004, HR 0.65, 95% confidence interval higher range < 1) (60.38 vs. 30.45 months) (Supplementary Table S5) (Figure 4G).

**Figure 5:**
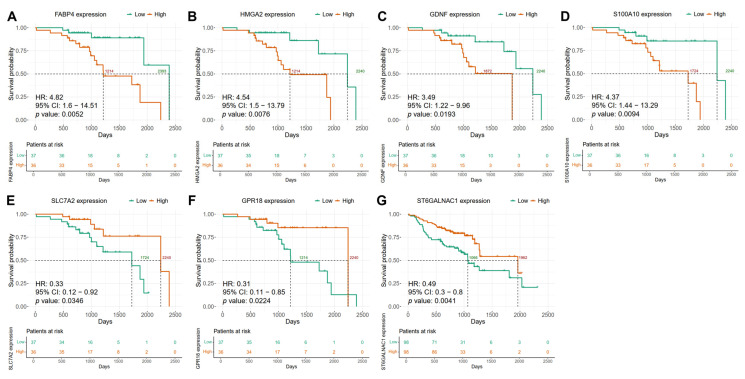
The association of overall survival and gene expression levels of (**A**) *FABP4*, (**B**) *HMGA2*, (**C**) *GDNF*, (**D**) *S100A10*, (**E**) *SLC7A2*, (**F**) *GPR18*, and (**G**) *ST6GALNAC1* was confirmed in the validation cohort using a univariate Cox analysis. The dotted line represents the 50% probability of survival.

The potential use of these seven candidate biomarkers for predicting clinical outcomes was confirmed using the validation cohort (HNSCC GSE65858 dataset from GEO) [27]. For the HPV-positive group, the association of high expression levels of *FABP4*, *GDNF*, *HMGA2*, and *S100A10* genes with poorer OS was confirmed (Figure 5). Elevated expression of *FABP4* was associated with shorter OS compared to the low-expression subgroup (1214 vs. 2393 days) (Figure 5A). Similar results were obtained when comparing high-expression and low-expression subgroups for *HMGA2* (1214 vs. 2240 days, respectively), *GDNF* (1872 vs. 2240 days, respectively), and *S100A10* (1724 vs. 2240 days, respectively) (Figure 5B–D). Furthermore, the protective effect of high expression compared with the low expression of *SLC7A2* and *GPR18* was also confirmed in the validation cohort (1724 vs. 2240 days and 1214 vs. 2240 days, respectively) (Figure 5E,F). Moreover, for the HPV-negative group we also confirmed the association of *ST6GALNAC1* high expression with better prognosis when compared with the low-expression subgroup (1962 vs. 1066 days, respectively) in the validation cohort (Figure 5G).

**Figure 6:**
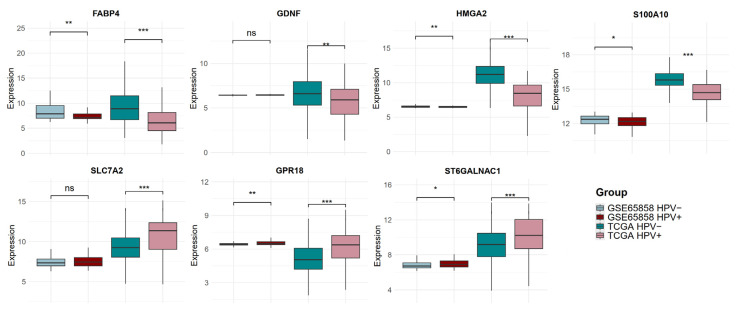
Gene expression of candidate biomarkers in discovery and validation cohorts. The expressions of *FABP4*, *GDNF*, *HMGA2*, *S100A10*, *SLC7A2*, *GPR18*, and *ST6GALNAC1* were evaluated in the validation cohort by dividing HNSCC into HPV-negative (HPV−) and HPV-positive groups. Asterisks represent a significant *p* value (*) < 0.05, (**) < 0.01, (***) < 0.001; ns, non-significant.

Subsequently, those genes related to OS in the validation cohort (GSE65858) were analyzed to evaluate their expression, dividing the cohort into HNSCC HPV-positive and HNSCC HPV-negative and comparing the candidate gene expression levels. Five out of the seven candidate genes were confirmed as differentially expressed in the validation cohort, *FABP4* (logFC −0.67, *p* value 0.002), *HMGA2* (logFC −0.06, *p* value 0.006), *S100A10* (logFC −0.17, *p* value 0.018), *GPR18* (logFC 0.11, *p* value 0.004), and *ST6GALNAC1* (logFC 0.14, *p* value 0.026). However, *GDNF* and *SLC7A2* were not differentially expressed in the validation cohort (Figure 6).

## 3. Discussion

It is widely known that HPV-positive and HPV-negative tumors have distinct identities and differ in their molecular biology and clinical course [17,28]. HPV-positive HNSCC, particularly OPSCC, is associated with a better prognosis and response to treatment [29,30,31,32,33]. Consistent with this, our analyses reveal that HPV-positive HNSCC patients have significant improvement in OS compared to HPV-negative patients. However, it has been shown that there is a subgroup of nearly 30% of OPSCC patients who, despite being HPV-positive, experience disease recurrence [34]. These findings suggest the existence of differences in molecular profiles within HPV-positive HNSCC patients that are associated with clinical outcomes. Studies focused on the analysis of these molecular profiles within HPV-positive patients have allowed the identification of cellular elements with potential use as biomarkers to stratify patients at higher risk of developing relapse or with poor OS [17]. The improvement in OS and progression-free survival (PFS) suggests that the de-escalation of treatment may be suitable for HPV-positive HNSCC patients [35,36,37]. Several clinical trials have evaluated a variety of dose de-escalation protocols, specifically for patients with HPV-related OPSCC [38,39,40]. Nevertheless, despite the generally good prognosis for HPV-related OPSCC, around 20% of the patients do not show an improved prognosis or response to treatment [36,40]. The determination of HPV positivity is thus becoming a key factor in the prognosis and treatment of patients with HNSCC. Traditional tools to assess HPV positivity include the detection of the viral genome by PCR, the expression of E6 and E7 viral oncogenes by RT-PCR, the *in situ* hybridization of E6 and E7 mRNA, and the expression of the surrogate marker p16 by immunohistochemistry in HNSCC biopsies. By using modern technologies such as next-generation sequencing, not only has HPV positivity been analyzed in HNSCC patients but also viral gene expression and the frequency of viral genome integration sites into the host genome [41]. Therefore, our discovery cohort (TCGA) includes only those patients with information on HPV positivity by different molecular techniques. We performed an exhaustive review of the literature using TCGA HNSCC cohort in addition to TCGA clinical annotation in order to classify samples with multiple complementary approaches. Only samples consistently identified as HPV-positive or -negative across all studies [24,25,26] were included, reducing the misclassification bias a limitation in many prior studies. This conservative strategy allows a greater robustness and certainty in the differential gene expression analysis between positive and negative tumors. Only these were then used for the subsequent analysis.

Previously, other studies have focused on elucidating the differences in expression profiles in HPV-positive vs. HPV-negative HNSCC to understand the altered molecular mechanisms in these tumors or to associate cellular processes that confer sensitivity to treatment in HPV-positive HNSCC. For instance, Hinić et al. (2022) [42] identified 1854 differentially expressed genes in HPV-positive vs. HPV-negative HNSCC. In agreement with our results, they found that ECM–receptor interaction, focal adhesion, PI3K-Akt signaling, cytokine–cytokine receptor interaction, hematopoietic cell lineage, protein digestion, and absorption pathways were altered in these types of tumors [42].

Genes associated with OS were identified in the discovery set in both HPV-positive and -negative HNSCC after dichotomized expression into high and low, according to the median. When DEGs of HPV-positive vs. HPV-negative tumors were merged with OS-related genes in either HPV-positive or -negative HNSCC, only 160 genes were significantly associated with OS in the HPV-positive group, while 222 genes were identified in HPV-negative tumors (Supplementary Table S4). After selecting genes with HR > 1, which also maintained an association with OS in a validation cohort dataset (GSE65858) and were not related to disease stage or alcohol history as confounders (when using a multivariate proportional hazard Cox model), six genes including *FABP4*, *GDNF*, *HMGA2*, *S100A10*, *SLC7A2*, and *GPR18*, were found to be significantly associated with clinical outcomes exclusively in HPV-positive HNSCC patients (Table 1). Meanwhile, only the *ST6GALNAC1* gene was significantly associated with OS in patients with HPV-negative HNSCC and not in HPV-positive tumors in both the discovery and validation cohorts, without any significant association with confounders in the multivariate Cox analysis. Notably, our analysis showed other genes associated with clinical outcome in patients with HPV-positive and HPV-negative HNSCC, which were not found to be differentially expressed between the two groups, which exhibited a significant risk value that were not considered in our study and which will require further attention to clarify their participation as prognostic biomarkers. The biological functions of the genes identified as prognostic biomarkers in this study, as well as their involvement in cancer and their impact on clinical outcome in HNSCC and other cancers, are described in Table 2.

Overall, our findings show that HPV-positive HNSCC are a heterogeneous group of tumors with differential molecular behaviors that impact the clinical outcome of the patients. It is important to mention that, although HPV-positive HNSCCs have traditionally been the group that responds best to treatment, around 30% of these patients experience a recurrence event [34]. This work provides insight into the molecular mechanisms that may underlie poor survival in a set of HPV-positive patients and proposes candidate biomarkers that could aid in identifying these high-risk individuals. Therefore, the search for biomarkers that identify HPV-positive patients who are at higher risk of death or recurrence becomes essential for providing patients with closer follow-up or, eventually, more appropriate therapy. In addition, the importance of de-escalating conventional treatment in a subset of HPV-positive patients to improve their quality of life should be considered. The bioinformatic analysis of transcriptomic databases from open repositories allows a clear approach of the molecular processes inherent to cancer and their association with the clinicopathological characteristics of patients with HNSCC, where the design of bioinformatic strategies is a critical point to successfully find accurate prognostic biomarkers. A separate analysis for HPV-positive and HPV-negative patients was performed and retained only those genes that were both differentially expressed and significantly associated with overall survival within each group. This stratified approach allows for the identification of group-specific prognostic biomarkers and may support the development of tailored clinical management strategies for HPV-positive and HPV-negative HNSCC patients. Moreover, we found biomarkers whose prognostic value was independent of tumor stage and alcohol history, suggesting potential utility for patient stratification even in early-stage disease. In this work, we present for the first time *FABP4*, *GDNF*, *HMGA2*, *S100A10*, *SLC7A2*, and *GPR18* as potential prognostic biomarkers exclusively for HPV-positive HNSCC and *ST6GALNAC1* only for HPV-negative HNSCC, which despite showing a significant prognostic value, need to be evaluated in different cohorts with characteristics inherent to the study population, in addition to the inclusion of other molecular techniques such as immunohistochemistry or PCR, which have shown great clinical utility. Our study centers on mRNAs, which are easier to validate in clinical settings than other RNA types. Moreover, many of the identified genes encode proteins that could be assessed post-surgery through immunohistochemical analysis, paving the way for potential protein-level biomarker validation. The prognostic biomarkers identified in this study show potential in translational medicine, allowing clinicians to make decisions aimed at improving treatments that impact patients’ quality of life. Furthermore, this study opens up new lines of research focused on identifying potential therapeutic targets.

## 4. Methods

### 4.1. Data Collection and Pre-Processing of HNSCC Transcriptomics

Clinical and gene expression data from RNA-seq profiling were obtained from the Firebrowse website [80] and TCGA Head and Neck Cancer cohort [81], respectively. A total of 500 HNSCC samples were included in our analysis and were classified into two groups: HPV-positive and HPV-negative HNSCC. To obtain a reliable transcriptional signature of HPV-positive HNSCCs for the prediction of clinical outcomes in patients, our analysis included only those HNSCC samples classified as HPV-positive according to the TCGA clinical annotation and three other independent analyses of samples from the same cohort, using RNA-seq, exome and whole-genome sequencing, as well as a reanalysis of the original sequencing data [24,25,26]. In total, 111 samples were classified as HPV-positive in at least one of the reviewed studies. However, only 61 samples shared HPV positivity by the different HPV detection methods and were classified as HPV-positive in all studies. Furthermore, all of the samples included in this study have RNA-seq data available. Similarly to the selection of HPV-positive cases, HPV-negative HNSCC samples were considered as such when they were typed as HPV-negative in the TCGA clinical annotation and all independent analyses, using different methods. In total, 409 samples were considered to be negative.

### 4.2. Data Normalization and Differential Gene Expression Analysis of HNSCC HPV-Positive vs. HPV-Negative HNSCC

**Figure 7:**
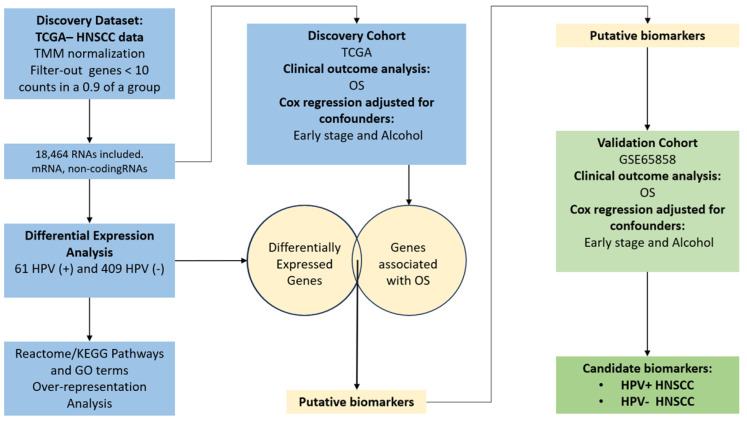
Bioinformatics workflow for the search for biomarkers related to clinical outcomes in patients with HPV-positive HNSCC. RNA-seq data was obtained from the TCGA HNSCC cohort. Samples were divided into two groups: HPV-positive (HPV+) and -negative (HPV−) HNSCC, according to the HPV status reported by TCGA and by other analyses carried out previously; only samples with concordant HPV status were included in this study. After pre-processing and normalization, 18,464 gene expression profiles were included in our study. We then performed a differential expression analysis considering the HPV status of HNSCC samples (HPV-positive vs. HPV-negative). The functions of differentially expressed genes (DEGs) were determined using over-representation analysis of GO terms, KEGG, and Reactome pathways. Gene expression levels were evaluated for their role in predicting clinical outcomes using a Cox proportional hazard model according to overall survival only in HNSCC HPV-positive patients. Genes with an impact on overall survival (OS), a 95% interval confidence (IC) > 1 (or <1 for protective effect), and a *p* value < 0.05, were evaluated in an independent dataset (GSE65858) of HPV-positive HNSCC patients. Then, gene expression, tumor stage (early vs. advanced), and alcohol consumption history were included in a multivariate Cox proportional hazard model. Genes that remained significantly associated with overall survival (*p* < 0.05) and were not significantly associated with the confounding variables (*p* > 0.05) were retained as candidate biomarkers. A similar approach was used for HPV-negative HNSCC patients.

A total of 66 HPV-positive and 409 HPV-negative samples were included in differential expression analysis (DEA). Genes with less than 10 counts per million (cpm) in 90% of the samples of at least one of the groups (HPV-positive or HPV-negative) were excluded from the differential expression analyses, as suggested for bulk sequencing [82]. Thus, the expression of 18,464 transcripts were included for differential expression analysis (DEA) (Figure 7) (Supplementary Table S6). For normalization and DEA, we used the DESeq2 package [82]. Counts were normalized using the *estimateSizeFactors* function, and then the differential gene expression analysis was performed. Differentially expressed genes (DEGs) were considered as such after a cut-off value of adjusted *p* value < 0.05 and a fold-change > |1|.

### 4.3. Function Annotation of Differentially Expressed Genes in HPV-Positive HNSCC

To identify the functions of DEGs, we performed an over-representation analysis of Gene Ontology (GO) terms and metabolic and signaling pathways using the Kyoto Encyclopedia of Genes and Genomes (KEGG) [83] and Reactome [84] databases. These analyses were performed using the ShinyGO webtool (version 0.8) [85], using the list of 2080 DEGs as input for the analyses, as well as the 18,464 genes used for DEA as background expressed genes.

### 4.4. Clinical Outcome Analysis in HPV-Positive HNSCC from TCGA and Validation Cohorts

Univariate Cox proportional hazard model and log rank test were performed with the survival package (version 3.5-7) [86] in R (version 4.3.1). We performed the survival analyses of HPV-positive and HPV-negative samples independently. All DEGs were evaluated individually in the analysis. The samples were divided into two groups (high expression and low expression) according to the median of each DEG expression level. A gene was considered relevant for overall survival if the 95% confidence interval of the HR was either >1 (indicating increased risk) or <1 (indicating a protective effect), and the *p* value for the log-rank test and univariate Cox proportional hazard model was <0.05. Subsequently, a multivariate Cox analysis was performed to evaluate the influence of additional clinicopathological factors on OS. Specifically, we included gene expression, disease stage, and alcohol consumption history in the same multivariate Cox proportional hazard model to determine whether gene expression remained significantly associated with overall survival after adjusting for these potential confounders. For this analysis, tumor stages I and II were grouped as early-stage HNSCC, while stages III and IV were considered advanced-stage. Regarding alcohol history, patients were categorized as either alcohol consumers or non-consumers. The discovery and validation cohorts were analyzed independently. Genes were considered robust predictors of OS if they met the following criteria: (i) 95% confidence interval of the HR > 1 or < 1, (ii) *p* value < 0.05, and (iii) no significant association with the assessed confounders (*p* value > 0.05). Genes showing a significant association with any of the confounding variables were excluded from further consideration. To create the Kaplan–Meier plots of the results, survminer (version 0.4.9) [87] and ggplot2 (version 3.5) [88] packages were used.

The GSE65858 HNSCC dataset [27] available in Gene Expression Omnibus (GEO) was used as a validation cohort for overall survival (OS) analyses, according to the above-mentioned strategy. The cohort includes 270 samples, 73 HPV-positive, 196 HPV-negative, and 1 with no HPV status Genotyping of the samples in this study was performed by detecting viral DNA using the reverse hybridization assay.

## Figures and Tables

**Table 1:** Confounder analysis shows no significant association of early stage or alcohol history with overall survival within HPV-positive (HPV+) or -negative (HPV−) HNSCC patients. Early-stage includes stages I and II, and stages III and IV are considered advanced-stage. The multivariate Cox proportional hazard model was performed using the survival package in R. (^†^) is not significant in the discovery cohort.

Discovery Cohort (TCGA)						
HNSCC	Gene	HR	95% IC	High Expression *p* Value	Early-Stage *p* Value	Alcohol History *p* Value
**HPV+**	*FABP4*	3.34	1.04–10.73	0.0426	0.3678	0.5836
	*GDNF*	3.41	1.07–10.83	0.0379	0.9975	0.8007
	*HMGA2*	3.35	1.08–10.37	0.0357	0.5047	0.9147
	*S100A10* ^†^	3.05	0.98–9.51	0.0538	0.6274	0.8405
	*SLC7A2*	0.32	0.1–0.99	0.0481	0.2619	0.9952
	*GPR18*	0.25	0.07–0.84	0.0253	0.4677	0.6414
**HPV**−	*ST6GALNAC1*	0.63	0.47–0.85	0.0027	0.073	0.8883
**Validation Cohort (GSE65858)**						
**HPV+**	*FABP4*	5.18	1.59–16.93	0.0065	0.9976	0.3013
	*GDNF*	3.19	1.12–9.05	0.0294	0.9975	0.8007
	*HMGA2*	3.96	1.3–12.1	0.0157	0.9976	0.7485
	*S100A10*	2.97	1.05–8.41	0.041	0.9975	0.7149
	*SLC7A2*	0.3	0.11–0.83	0.0209	0.9975	0.7638
	*GPR18*	0.34	0.12–0.95	0.0389	0.9975	0.8128
**HPV−**	*ST6GALNAC1*	0.55	0.33–0.9	0.018	0.0744	0.8922

**Table 2:** Functional description of HPV-positive and HPV-negative HNSCC potential biomarkers found in this study.

Biomarkers	Function	Alterations in Cancer	Clinical Outcome	References
*FABP4* (Fatty Acid Binding Protein 4)	Fatty acid binding proteins (FABPs) are intracellular lipid chaperones that reversibly bind hydrophobic ligands and coordinate lipid responses in cells. *FABP4*, also known as A-FABP, is primarily expressed in adipocytes, macrophages, and dendritic cells.	*FABP4* expression in tumor-associated macrophages promotes breast cancer progression. Angiogenesis, growth, and metastasis in ovarian tumor xenografts were significantly inhibited by suppressing endothelial *FABP4* expression.	High *FABP4* expression is associated with poor OS and relapse-free survival (RFS) in patients with lung, gastric, and colorectal cancers. Loss of *FABP4* expression was correlated with poor prognosis in bladder cancer.	[43,44,45,46,47,48,49,50]
*S100A10* (S100 Calcium Binding Protein A10)	*S100A10* belongs to the S100 family of calcium-binding proteins and constitutes the largest subgroup of EF-hand proteins. *S100A10*, also known as p11, regulates plasminogen activation and plasmin generation.	*S100A10* was shown to be positively associated with metastasis, blood vessel development, EMT, hypoxia, and invasion in HNSCC.	High expression is linked to a worse prognosis in patients with hepatocellular carcinoma, pancreatic adenocarcinoma and glioma. Low *S100A10* gene expression was associated with good OS in patients with HNSCC.	[51,52,53,54]
*HMGA2* (High Mobility Group AT-Hook 2)	*HMGA2* is a nonhistone chromatin factor that binds to AT-rich DNA sequences affecting chromatin structure, thereby enhancing or suppressing the activity of many transcription factors. HMGA proteins regulate embryonic development, stem cell maintenance, cellular senescence, and tumorigenesis.	*HMGA2* facilitates triple-negative breast cancer metastasis by transcriptionally activating matrix metalloproteinases. Specifically, *HMGA2* induced chromatin conformation changes to enhance MMP transcriptional activity. *HMGA2* is overexpressed in HNSCC and induces the expression of genes associated with the cancer stem cell phenotype. *HMGA2* promotes malignant progression of HNSCC and the acquisition of CSC properties by directly regulating Snai2 expression.	Higher *HMGA2* expression was associated with poor OS in different types of cancer, including HNSCC. High gene and protein expression were associated with decreased OS and disease-free survival (DFS) in HNSCC patients. *HMGA2* expression was an independent predictor of OS and DFS in oral cancer and HNSCC	[55,56,57,58,59,60,61,62]
*GDNF* (Glial Cell Derived Neurotrophic Factor)	*GDNF* is a member of the *GDNF* ligand (GFL) family. It binds to *GDNF* receptor-α1 (GFRα1) and its coreceptor, RET. This growth factor has been implicated in neuronal differentiation and survival of neurons	In HNSCC, *GDNF* expression was associated with increased perineural invasion and lymphatic metastasis by increasing PD-L1 expression through the JAK2-STAT1 signaling pathway.	High expression was related to decreased overall survival in HNSCC patients. *GDNF* positivity correlated with poorer OS and PFS, regardless of HPV status, in HNSCC patients. The presence of the *GDNF* protein in stroma was associated with a significantly higher risk of death only in the HPV-negative HNSCC patients. Higher *GDNF* gene expression was associated with better clinical outcomes in HPV-negative HNSCC patients.	[63,64,65,66]
*SLC7A2* (Solute Carrier Family 7 Member 2)	*SLC7A2* is a membrane protein that facilitates the cellular transport of cationic amino acids, including lysine and L-arginine.	*SLC7A2* is highly expressed in normal tissues compared to HNSCC. The overexpression of *SLC7A2* promotes apoptosis in HNSCC cells while effectively inhibiting their growth, proliferation, and metastasis.	High levels of *SLC7A2* correlated with prolonged OS in HNSCC patients, and this survival difference was even more prominent in HPV-positive cases.	[67,68,69]
*GPR18* (G Protein-Coupled Receptor 18)	*GPR18* is an atypical cannabinoid receptor capable of binding N-arachidonoyl glycine (NAGly) and resolvin D2 (RvD2), anti-inflammatory compounds that could contribute to limiting tumor spread.	*GPR18* is overexpressed in melanoma metastatic sites, is constitutively active, and inhibits apoptosis.	Increased expression of *GPR18* correlated with an increase in OS of patients with HNSCC.	[70,71,72,73,74]
*ST6GALNAC1* (ST6 N-Acetylgalactosaminide Alpha-2,6-Sialyltransferase 1)	The ST6GalNAc-I enzyme is a glycosyltransferase capable of adding sialic acid at the 2,6 linkage to GalNAc linked to serine or threonine, thus creating the STn epitope.	High levels of *ST6GALNAC1* in ovarian cancer cells promote cell proliferation, migration, invasion, self-renewal capacity, and tumorigenicity. In breast cancer cells, *ST6GALNAC1* expression significantly enhanced cell migration and invasion, and it was found to directly induce the EMT signaling pathway.	*ST6GALNAC1* positivity in stage III and IV colorectal cancer samples is significantly associated with short OS. Low *ST6GALNAC1* gene expression is associated with poor OS, RFS, and post-progression survival (PPS) in colorectal cancer patients.	[75,76,77,78,79]

## Data Availability

The original contributions presented in this study are included in the article/Supplementary Materials. Further inquiries can be directed to the corresponding authors.

## Supplementary Materials

The following supporting information can be downloaded at: https://www.mdpi.com/article/10.3390/ijms26125894/s1.
